# Prevalence of Glomerulopathies in Canine Mammary Carcinoma

**DOI:** 10.1371/journal.pone.0164479

**Published:** 2016-10-20

**Authors:** Leandro Z. Crivellenti, Gyl E. B. Silva, Sofia Borin-Crivellenti, Rachel Cianciolo, Christopher A. Adin, Márcio Dantas, Denner S. dos Anjos, Mirela Tinucci-Costa, Aureo E. Santana

**Affiliations:** 1 Department of Veterinary Clinic and Surgery, Franca University (UNIFRAN), Franca, Brazil; 2 Department of Veterinary Clinic and Surgery, São Paulo State University (UNESP), Jaboticabal, Brazil; 3 Department of Renal Pathology, Faculty of Medicine of Ribeirão Preto, Universidade do Estado de São Paulo (USP), São Paulo, Brazil; 4 Department of Veterinary Biosciences, College of Veterinary Medicine, The Ohio State University, Columbus, United States of America; 5 Department of Clinical Sciences, College of Veterinary Medicine, NC State University, Raleigh, United States of America; 6 Department of Internal Medicine (Division of Nephrology), Faculty of Medicine of Ribeirão Preto, Universidade do Estado de São Paulo (USP), São Paulo, Brazil; Emory University Department of Medicine, UNITED STATES

## Abstract

The incidence and prevalence of paraneoplastic glomerulopathy, especially associated with carcinoma, are a matter of debate and the causal link between cancer and glomerular diseases remains unclear. The aim of this study was to evaluate renal biopsies of selected bitches with spontaneous mammary gland carcinoma. We hypothesized that dogs with mammary carcinomas would show histologic evidence of glomerular pathology. A prospective study was performed in dogs with naturally occurring mammary carcinoma that were undergoing tumor resection and ovariohysterectomy. We evaluated renal biopsies of 32 bitches with spontaneous mammary gland carcinoma and 11 control dogs without mammary gland neoplasia. Samples were obtained from the left kidney and the biopsy material was divided for light microscopy (LM), immunofluorescence (IF) and transmission electron microscopy (TEM). Light microscopy abnormalities were identified in 78.1% of dogs with mammary carcinoma (n = 25) and in none of the dogs in the control group. Focal glomerular mesangial matrix expansion was the most common alteration (n = 15, 60.0%), but mesangial cell proliferation (n = 9, 36.0%) and focal segmental glomerulosclerosis (n = 9, 36.0%), synechiae (n = 7, 28.0%), and globally sclerotic glomeruli (n = 6, 24.0%) were also frequent in dogs with malignancy. Immunofluorescence microscopy revealed strong IgM staining was demonstrated in 64.3% (n = 18) of carcinoma dogs. Transmission electron microscopy from dogs with carcinoma revealed slight changes, the most frequent of which was faint sub-endothelial and mesangial deposits of electron-dense material (78%). Mesangial cell interpositioning and segmental effacement of podocyte foot processes were identified in some specimens (45%). Changes in the glomerulus and proteinuria are common in dogs with naturally occurring mammary carcinoma and this condition appears to provide an excellent large animal model for cancer-associated glomerulopathy in humans.

## Introduction

Breast cancer is considered one of the most common malignancies affecting humans and is therefore an area of intense research [1]. Naturally occurring mammary carcinoma is also seen with a high incidence in bitches, and dogs might serve as an excellent model for human disease [2]. While much information has been gathered about the biological behavior of breast cancer, the pathophysiology of solid tumor-associated glomerulopathies remains obscure [3,4,5]. Glomerular pathology associated with malignancy is not uncommon in human patients [6] and affected patients have been reported to develop renal failure, paraneoplastic glomerulopathy and sometimes nephrotic syndrome [7]. Furthermore, the clinical entity of glomerulopathy remains poorly understood and no accepted experimental model of cancer-associated glomerulopathy has been evaluated [8]. In particular, the rodent model of carcinoma is poorly correlated with the human model because rats often die before developing proteinuria [3].

The incidence and prevalence of paraneoplastic glomerulopathy, especially associated with carcinoma [9], are a matter of debate and the causal link between cancer and glomerular diseases remains unclear [6,7]. Membranous glomerulonephropathy (MGN) is the most frequent glomerular lesion identified in association with advanced cases of solid tumors [4,6,8] but minimal change disease (MCD), focal segmental glomerulosclerosis (FSGS), membranoproliferative glomerulonephritis (MPGN) [6,8] and thrombotic microangiopathy (TMA) [10] also have been reported to be associated with breast cancer. Thus, determining the prevalence of glomerulopathies in dogs with mammary carcinoma could help elucidate pathogenesis of lesion development and could support future use of the dog as a model for human disease. The aim of this study was to evaluate renal biopsies of 32 selected bitches with spontaneous mammary gland carcinomas and 11 control dogs without mammary gland neoplasia using light microscopy (LM) and immunofluorescence (IF) and transmission electron microscopy (TEM). We hypothesized that dogs with mammary carcinomas would show histologic evidence of glomerular pathology at a higher rate than in the control population.

## Materials and Methods

The following study was approved by the Veterinary Ethics Committee of UNESP—Univ Estadual Paulista, Jaboticabal, Brazil (0118662/11). A prospective study was performed in dogs attended at the Veterinary Teaching Hospital of UNESP with naturally occurring mammary carcinoma that were undergoing tumor resection and ovariohysterectomy. The animals included were client-owned and written owner consent was obtained for all dogs prior to inclusion in the study. Animals with a histological diagnosis of mammary gland carcinoma and without evidence of metastasis on chest radiographs, abdominal ultrasound and histopathological analysis of regional lymph nodes were selected for this study. Dogs with other types of neoplasms or ulcerated neoplasms, azotemia at time of diagnosis, or concurrent endocrinopathies were excluded from the study. Presence of proteinuria and hypertension were not inclusion or exclusion criteria. Our control population consisted of age-matched intact female dogs presenting for sterilization surgery with no evidence of mammary neoplasia, allowing us to minimize the effect of age or hormone-related changes on interpretation of results. The animals receive as premedication acepromazine plus morphine (0.05 and 0.2 mg/kg, respectively) mixed in same syringe injected intramuscularly; anesthetic induction was performed with propofol (5 mg/kg) intravenously (IV) followed by endotracheal intubation; anesthesia was maintained with isoflurane and IV fentanyl (0.005 mg/kg). Postoperative analgesia included the IV injection of meloxicam (0.1 mg/kg), metamizole (25 mg/kg) and tramadol (2 mg/kg).

Urine and blood samples were collected in 1 hour before ovariohysterectomy (control group) or ovariohysterectomy with mastectomy (carcinoma group). Complete blood count (CBC), biochemical profile (creatinine, urea, ALT, ALP, phosphorus, calcium, albumin, total protein, sodium and potassium), urinalysis and urine protein/creatinine ratio (UPC) were recorded for each dog. Both serum and urine were evaluated using SDS-PAGE electrophoresis. Serum IgG and IgA were visualized by comassie blue (Sigma-Aldrich). After centrifugation of 10 mL of urine supernatant was transferred to tubes to perform biochemical and urine proteins were fractionated by SDS-PAGE using 10.4% polyacrylamide with markers (a-lactalbumin: 14.2 kDa; trypsin inhibitor from soybean: 20.0 kDa; trypsinogen from bovine pancreas: 24 kDa; carbonic anhydrase from bovine erythrocytes: 29 kDa; glyceraldehyde-3-phosphate dehydrogenase from rabbit muscle: 36 kDa; ovalbumin: 45 kDa; albumin: 66 kDa; phosphorylase b: 97 kDa; β-galactosidase from *E*. *coli*: 116 kDa; myosin from rabbit muscle 205 kDa). All markers were from SigmaMarker, USA.

An open renal biopsy was performed in all dogs at the time of ovariohysterectomy. Samples were obtained from the left kidney using an 18, 16 or 14 gauge (G) semi-automated cutting needle (M.D.L Aghi specialli e componenti medicalli—Italy) and the biopsy material was divided for LM, IF and TEM.

Bouin-fixed samples were used for LM, and sections were cut at 2–3 μm thickness. Four different stains were used to evaluate the kidney: hematoxylin and eosin (HE), periodic acid Schiff (PAS), Jones methenamine silver (JMS), and Masson´s trichrome (TRI). When amyloid deposition was suspected, Congo Red staining was also performed on sections of 6 μm thickness. Two samples were analyzed in LM (> 10 glomeruli, 23.8±14.2).

For direct IF, Michel’s solution was used to transport the sample. Fresh unfixed renal specimens were washed with PBS, and then were embedded in optimal cutting temperature (OCT) compound. Samples were then snap-frozen in liquid nitrogen. Subsequently, 3–4 μm thick sections were cryosectioned and fixed with acetone for 15 minutes. After washing twice with PBS, the slides were incubated with polyclonal FITC-labeled goat anti-dog IgA, IgG, IgM, complement C3 antibodies (Bethyl Laboratories). Immunofluorescence examination was classified as granular or linear, and included the localization of the deposit (mesangium, capillary walls, tubules and blood vessels), distribution (focal, diffuse, segmental or global) and intensity. Immuno-labeling was graded using a semi-quantitative scale, ranging from 0, trace, 1+, 2+, and 3+.

All paraffin and frozen sections were examined by a physician nephropathologist who was blinded to the carcinoma status. The renal histological diagnoses were classified according to the World Health Organization’s classifications of human glomerular diseases as used in other studies [11–14]. Histopathologic evaluation of the mammary tumors was performed separately by a veterinary pathologist according to the classification and grading of canine mammary tumors as previously reported [15] to prevent bias in interpretation of renal samples. Total volume of the neoplasm was measured by π/6 x length x width x height formula [16].

Descriptive statistics and frequency distribution of variables were evaluated in each group. Normally distributed data were tested using a student’s *t* test. Immunofluorescence score data was not normally distributed and statistical comparisons were performed using Kruskal-Wallis analysis, followed by the Dunns’ post-hoc test. Significance was set at p<0.05. Associations between histological grade of the malignancy, number of tumors and tumor size on renal alterations (subjectively graded) and urinary and serum parameters were evaluated using a Pearson correlation. Calculations were performed using JMP^®^ software.

## Results

No significant changes were noted in CBC in either group. On the biochemistry panel, dogs with mammary gland carcinoma had significant increases in the following parameters compared to control dogs: total protein (6.04±1.15 and 7.22±0.95; *P* = 0.0084), globulins (2.76±0.66 and 4.27±1.16; *P* = 0.0117), transferrin (29.0±11.4 and 39.6±28.6; *P* = 0.0558) and haptoglobin (43.9±37.4 and 139.1±91.4; *P* = 0.0013). Globulin levels were positively correlated with tumor size (volume) (*r* = 0.21; *P* = 0.0087). Electrophoresis serum IgG and IgA were not different between groups. Systolic blood pressure was within normal limits (< 160 mmHg) in all animals.

The number of mammary nodules ranged from 1 to 10 (3.7±2.8) and total volume burden was 0.04 to 767.1 cm^3^ (109.3±199.4 cm^3^). The vast majority of carcinomas were classified as low-grade malignancies (n = 27, 84.4%). Intermediate grade and high grade carcinomas were identified in two (6.2%) and three (9.4%) bitches, respectively. Among the 32 bitches, 21 (65.6%) showed just one histopathological type of carcinoma and 11 (34.4%) had two types of carcinoma in the same animal. Tubular simple type carcinoma was observed in 19 bitches, tubular complex carcinoma in 13 bitches, malignant mixed carcinoma in 4 bitches, papillary in 3 bitches, and solid carcinoma in 3 bitches.

### Proteinuria

Urinary creatinine protein ratio (UPC) was significantly higher in dogs with mammary carcinoma than in age-matched controls (0.40±0.52 vs 0.11±0.08, *P*< 0.0001). No animals in the control group had proteinuria, while 10 dogs in the carcinoma group (31.25%) were proteinuric (UPC> 0.5). No dogs had more than 2.5 of protein in the urine prior to surgery. Urinary protein electrophoresis demonstrated the presence of low (10–50 kDa), medium (60–80 kDa) and high molecular weight (>80 kDa) proteins, consistent with both glomerular and tubular alterations in the carcinoma group.

### Light microscopy

**Fig 1:**
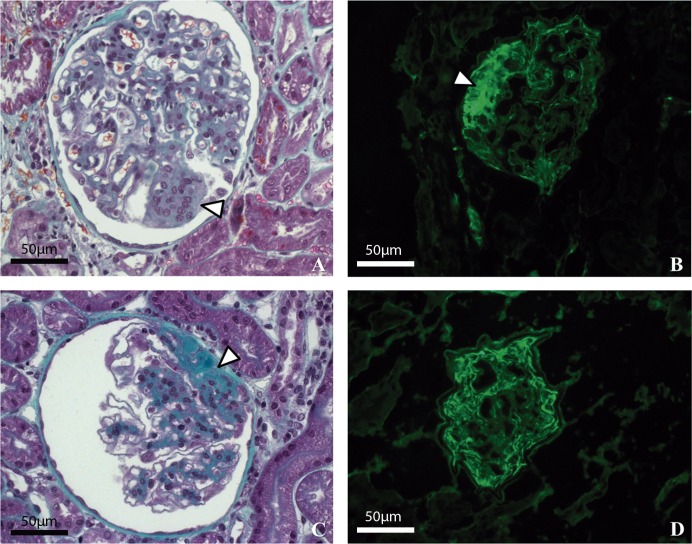
Microscopic findings in 2 dogs with mammary carcinoma. (A) Focal segmental glomerulosclerosis and mesangial cell proliferation (arrowhead). Masson’s trichrome (B) Moderate diffuse granular positivity for IgM in mesangium associated with trapping in a sclerotic segment (arrowhead). (C) Segmental sclerosis lesion with synechiae (arrowhead). Masson´s trichrome (D) Strong diffuse granular positivity for IgM in mesangium with some extensions along the capillary loop. Magnification x400.

LM abnormalities were identified in 25 dogs (78.1%) from the carcinoma group, but most findings were subtle. Focal glomerular mesangial matrix expansion was the most common alteration (n = 15, 60.0%), but mesangial cell proliferation (n = 9, 36.0%) and FSGS (n = 9, 36.0%), synechiae (n = 7, 28.0%), globally sclerotic glomeruli (n = 6, 24.0%), mild to moderate interstitial fibrosis and tubular atrophy (IFTA) (n = 5, 20.0%) were frequent (Fig 1A and 1C). Five bitches (20.0%) had hydropic degeneration of tubular epithelial cells. Furthermore, other abnormalities were found as hyaline arteriolosclerosis (n = 3, 12.0%), ischemic glomeruli (n = 3, 12%) and hemosiderin deposits (n = 2, 8.0%). Microscopic alterations in the glomeruli were positively correlated with mammary tumor volume (*r* = 0.56; *P* = 0.0008). No LM abnormalities were identified in the age-matched control group. In one case of carcinoma group, the silver stain showed sparse surface projections (spikes) along subepithelial surface of the glomerular basement membrane. Although, this histopathological feature is characteristic of membranous nephropathy, only weak IgA and strong IgM staining was observed on immunofluorescence.

### Immunofluorescence

From the 43 kidney biopsies, 3 dogs with carcinoma had no glomeruli available for IF evaluation. The numbers of dogs with positive immunolabeling for IgG, IgM, IgA and C3 are shown in Table 1.

**Table 1:** Number of dogs that had positive IF labeling with antibodies against canine IgG, IgM, IgA and C3.

	Control (n = 11)		Neoplasia (n = 29)		*p*
	positive IF	Intensity median (min-max)	positive IF	Intensity median (min-max)	
**IgM**^a^	3	0 (0–0.5)	28	2 (0–3)	<0.0001
**IgG**^b^	0	0 (0–0)	5	0 (0–2)	0.125
**IgA**^c^	0	0 (0–0)	7	0 (0–2)	0.015
**C3**^d^	0	0 (0–0)	5	0 (0–2)	0.062

^a^IgM: immunoglobulin M; IgG

^b^: immunoglobulin G; IgA

^c^: immunoglobulin A; C3

^d^: complement C3

In the mammary carcinoma group, 28 samples stained positively for IgM (96.6%). Of these, fourteen stained only for IgM (50.0%), three for IgM/IgG (10.7%), four for IgM/IgA (14.3%), four for IgM/C3 (14.3%), two for IgM/IgA/C3 (7.2%) and one for IgM/IgG/IgA (3.6%). Strong IgM staining was demonstrated in 64.3% (n = 18) of carcinoma dogs, moderate staining in 28.6% (n = 8) and weak staining in 7.1% (n = 2) (Fig 1B and 1D). Immunoglobulins were distributed in a granular pattern in the mesangium in 20 cases (71.4%).

There was extension to peripheral capillary loops in 7 cases (25.0%) and one in the vascular pole (3.6%). Three control animals (27.3%) were graded as trace to 1+ staining for IgM, with all other immunoreactants being negative.

Moderate to strong granular staining for IgG, and IgA in the mesangium and in capillary walls were recorded in 5 dogs (50%) with mammary neoplasia. Moderate linear C3 staining was observed in 4 dogs along tubular basement membranes.

### Transmission electron microscopy

**Fig 2:**
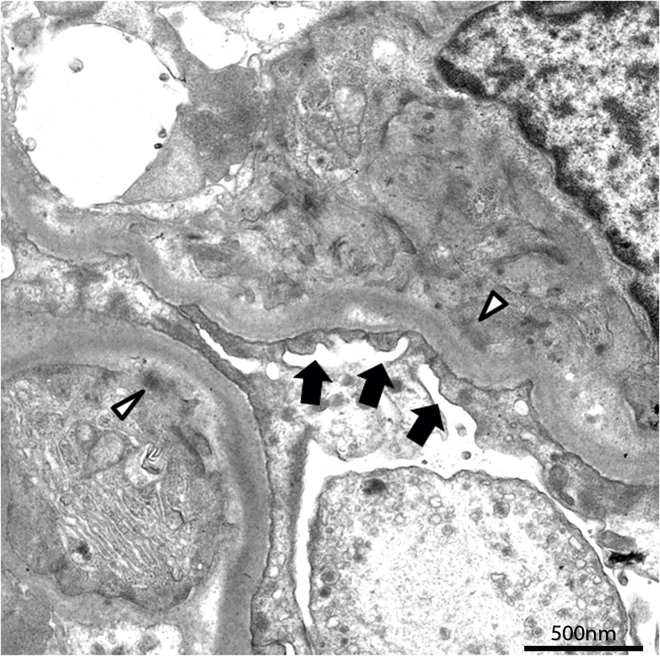
Ultrastructural changes observed in dogs from the carcinoma group. Weakly electron-dense mesangial deposit in upper capillary loop (open arrowhead) and effacement of the podocyte foot processes was often seen (black arrows). Original magnification, x120000.

From the 43 kidney biopsies, 7 dogs with carcinoma had no glomeruli available for TEM evaluation. Transmission electron microscopy of renal biopsies from dogs with carcinoma revealed slight changes, the most frequent of which was faint sub-endothelial and mesangial deposits of electron-dense material (n = 19, 76%). Sub-epithelial deposits of electron-dense material and mild capillary loops duplication of the glomerular basement membrane was observed in one case (4%). Mesangial cell interpositioning and segmental effacement of podocyte foot processes were identified in some specimens (n = 11, 44%) (Fig 2).

## Discussion

The results of our current study provides strong evidence that dogs with naturally occurring mammary carcinoma have an increased risk for development of subclinical proteinuria and glomerular lesions, similar to those described in humans with early stage neoplasms and subclinical glomerular disease [17]. Mammary carcinoma in dogs was associated with increased UPC and approximately one-third of the dogs in the carcinoma group were proteinuric (UPC>0.5). The high incidence of protein loss was accompanied by structural lesions on LM and TEM that are consistent with glomerular injury. Passive trapping of immunoglobulin deposits was common, with 28 of 29 dogs showing positive staining for IgM. While dogs in the carcinoma group had evidence of hyperglobulinemia, there was no difference in carcinoma vs control with respect to serum IgG and IgA. When examined overall, these data suggest that dogs in the carcinoma group had higher levels of IgM in their serum resulting in more passive trapping than in the control group (IgM was not evaluated in your study). Interestingly, our data showed evidence of an association between tumor volume and microscopic abnormalities, suggesting that glomerular injury progresses in coordination with increasing tumor burden.

In our study, we selected only animals without azotemia, ulcerated mammary tumors or gross evidence of metastases in order to avoid glomerular alterations from bacterial contamination and/or metastatic disease. As expected, proteinuria was not seen in any of the control dogs and IF staining was weak and rare when compared to the carcinoma group.

Since 1966 the interaction between cancer and glomerular disease has been described, with higher prevalence of glomerular diseases in patients with malignancy compared to a reference population [18]. However, no randomized-controlled study has been performed to evaluate the prevalence of renal alterations. To date, the most well-recognized and well-established associations with glomerular disorders have been in people with hematopoietic cancers [19] and solid tumor carcinomas [8]. One group has used a rat model (nude rat F344/NJcl-run) to study cancer related glomerulopathy [7]. While these animals developed proteinuria and decreased creatinine clearance over time, the use of an immunodeficient animal makes it difficult to extrapolate these results to the human cancer patient. However, findings in our current study suggest that the use of the dog as a naturally occurring model of tumor-associated glomerulopathy might be an excellent alternative.

Several factors have been suggested to contribute to the development of glomerular lesions in animals and humans with cancer, including decreased blood flow to the kidney [7], injury induced by products of the tumor cells [6] and deposition of immune complexes [4,17,20]. Our data suggest that higher globulin concentration (especially acute phase proteins) and the positive correlation with carcinoma size corroborate that abnormal products may be produced as an immune reaction to the solid tumor; and they might be present even in early stages of mammary carcinoma and these products can contribute to renal injury.

Membranous features as subepithelial deposits, and duplication of the glomerular basement membrane may occur in other glomerular diseases both in humans and dogs [21, 22, 23], but the characteristics of membranous nephropathy were reported in just one animal in current study (4%). Sparse or segmental deposits are seen in MGN in two instances in people: when biopsies are performed wither early in the course of disease or following resolution of the glomerular lesion. Previous reports in humans with cancer describe the incidence of membranous glomerulonephropathy being as low as 1% to as high as 22% [4,5,6,8]. In our study, dogs were biopsied early in the course of neoplastic disease and before the development of systemic metastases. It is possible that the development of membranous nephropathy occurs at later disease stages with chronic exposure to tumor-associated antigens or greater tumor burden. LM alterations similar to those in our study were found in humans with various early stage neoplasia in a previous study [17]. Interestingly, these lesions were not seen in the rat experimental carcinoma model [7] providing further evidence that the short time of cancer exposure and hereditary immunodeficiency in these rats may have altered the condition from that seen in humans or dogs with naturally occurring disease.

IgM nephropathy is a condition in humans that has been defined by > 2+ IgM staining with or without concurrent staining for C3. While many of the dogs described in this report showed strong positive staining for IgM and this was associated with mesangial electron-dense deposits, IgM nephropathy has not been described in dogs, and even in humans, the diagnosis is somewhat controversial [24,25,26]. While Non-specific deposits (i.e. trapping) may explain IgM-positive staining in sclerotic regions, and this would not be accompanied by simultaneous complement factors. [14]. IgM is a large glycoprotein (880 to 941 kD) and can be trapped nonspecifically in glomeruli, and even passive trapping may be responsible for some forms of glomerulonephritis that are associated with mesangial or subendothelial deposits [27]. Positive IF reactions for IgM was dominant in the current study and were different from other studies in rats [7] and humans [17,19], which found primarily IgG deposits due to deposition of circulating immune complexes [19]. This difference in pathophysiology could be explained by the fact that serum IgG and IgA were not increased in the canine carcinoma group. Alternatively, it might be related to the difference in stage of disease (early vs late) or the species being examined.

Despite the fact that rare subendothelial deposits (a feature of MPGN) were identified in some dogs of the carcinoma group, these animals did not show LM abnormalities consistent with MPGN, as has been reported in experimental carcinoma in rats [7] and in humans with early stage neoplasms [17]. However, most of our findings were diagnostic for FSGS and / or focal mesangial cell proliferation. This also differs from other studies in which renal biopsies were performed to evaluate clinical proteinuria [5,6,8,28]. Although minimal change disease is usually associated with malignancies in humans, it was not observed in our cohort of dogs with carcinoma [7,28]. In our study, we found that bitches with mammary carcinoma had evidence of segmental podocyte foot process fusion which likely contributed to proteinuria. Prior to our study, studies in women with breast cancer suggested that carcinoma could cause histopathologic lesions in the kidney [4,8], but that it was not sufficient to cause significant proteinuria [4]. Similarly, we suggest that in dogs with naturally occurring mammary carcinoma, subtle changes are frequent and are linked to subclinical proteinuria.

In conclusion, changes in the glomerulus and proteinuria are common in dogs with naturally occurring mammary carcinoma and this condition appears to provide an excellent large animal model for cancer-associated glomerulopathy in humans. Studying this condition and elucidation of the pathogenesis of the glomerular disease would allow earlier intervention in the course of renal injury, and potentially prevent more serious chronic glomerular disease in animals and human beings with carcinoma.

## Supporting Information

S1 Fig Microscopic findings in 2 dogs with mammary carcinoma. (A) Focal segmental glomerulosclerosis and mesangial cell proliferation (arrowhead). Masson’s trichrome (B) Moderate diffuse granular positivity for IgM in mesangium associated with trapping in a sclerotic segment (arrowhead). (C) Segmental sclerosis lesion with synechiae (arrowhead). Masson´s trichrome (D) Strong diffuse granular positivity for IgM in mesangium with some extensions along the capillary loop. Magnification x400.
(TIF)

S2 Fig Ultrastructural changes observed in dogs from the carcinoma group. Weakly electron-dense mesangial deposit in upper capillary loop (open arrowhead) and effacement of the podocyte foot processes was often seen (black arrows). Original magnification, x120000.
(TIF)

S1 Table Number of dogs that had positive IF labeling with antibodies against canine IgG, IgM, IgA and C3. (PDF)
