# Supplementary figures and images for: Prevalence of Glomerulopathies in Canine Mammary Carcinoma
Source: PLoS One. 2016 Oct 20;11(10):e0164479. doi: 10.1371/journal.pone.0164479 (PMC5072677; doi:10.1371/journal.pone.0164479)

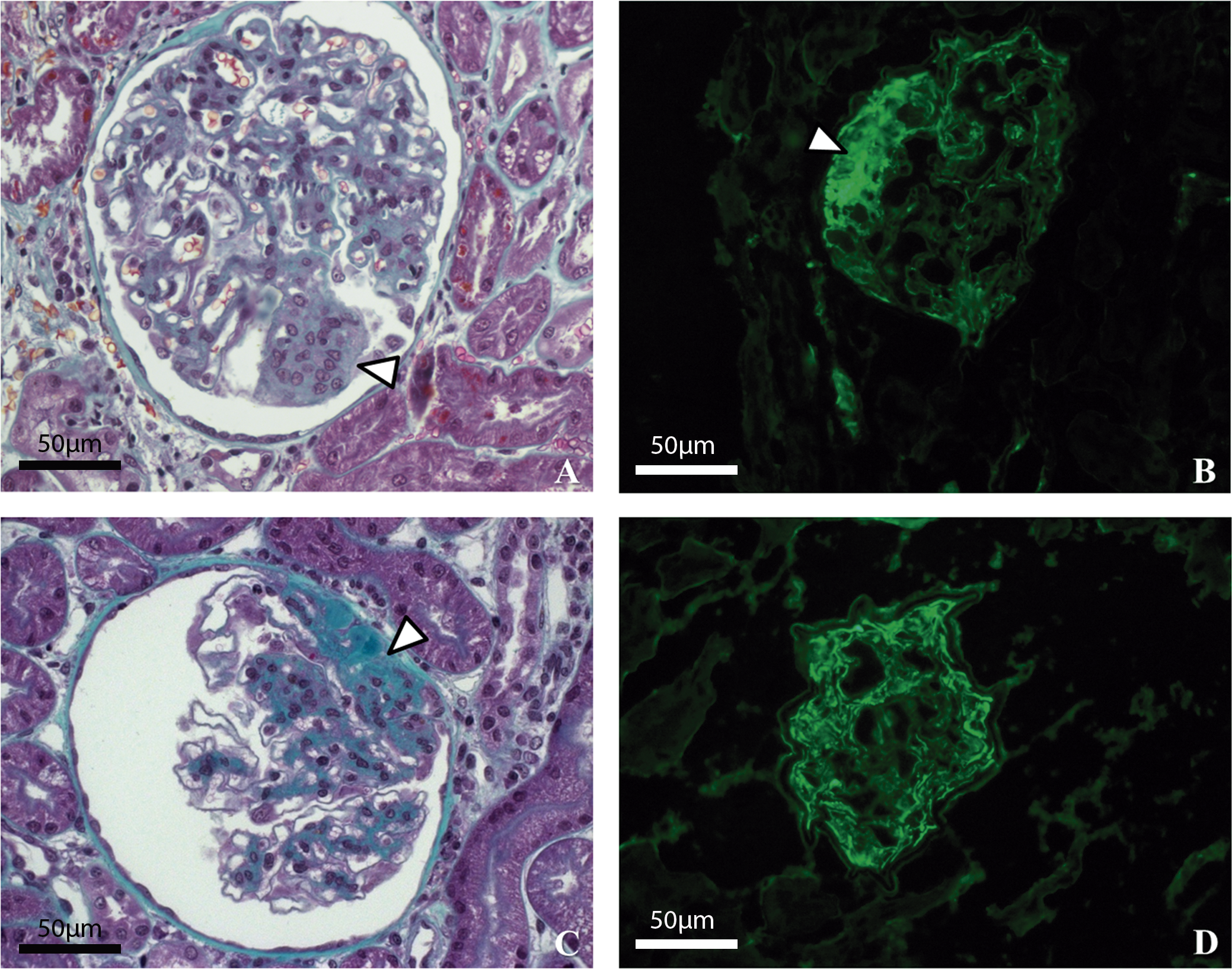

Supplement: S1 Fig — (A) Focal segmental glomerulosclerosis and mesangial cell proliferation (arrowhead). Masson’s trichrome (B) Moderate diffuse granular positivity for IgM in mesangium associated with trapping in a sclerotic segment (arrowhead). (C) Segmental sclerosis lesion with synechiae (arrowhead). Masson´s trichrome (D) Strong diffuse granular positivity for IgM in mesangium with some extensions along the capillary loop. Magnification x400. (TIF) [file pone.0164479.s001.tif]

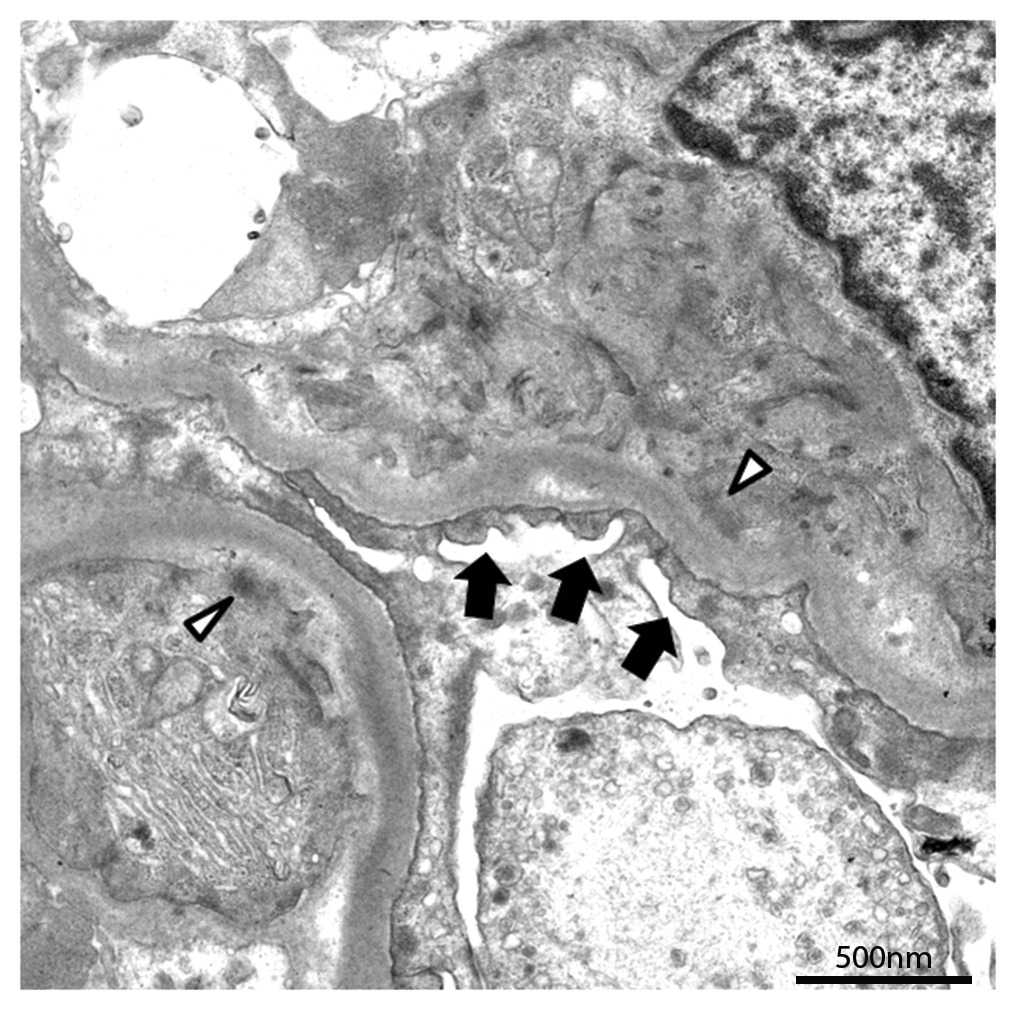

Supplement: S2 Fig — Weakly electron-dense mesangial deposit in upper capillary loop (open arrowhead) and effacement of the podocyte foot processes was often seen (black arrows). Original magnification, x120000. (TIF) [file pone.0164479.s002.tif]
