# Supplementary material for: Prevalence of Glomerulopathies in Canine Mammary Carcinoma
Source: PLoS One. 2016 Oct 20;11(10):e0164479. doi: 10.1371/journal.pone.0164479 (PMC5072677; doi:10.1371/journal.pone.0164479)
Supplement: S1 Table — (PDF) [file pone.0164479.s003.pdf]

S1 Table 1. Number of dogs that had positive IF labeling with antibodies against canine IgG, IgM, IgA and C3.

	**Control (n =11)**		**Neoplasia (n =29)**		***p***
	**positive IF**	**Intensity median (min-max)**	**positive IF**	**Intensity median (min-max)**	
**IgM**[a]	3	0 (0-0.5)	28	2 (0-3)	<0.0001
**IgG**[b]	0	0 (0-0)	5	0 (0-2)	0.125
**IgA**[c]	0	0 (0-0)	7	0 (0-2)	0.015
**C3**[d]	0	0 (0-0)	5	0 (0-2)	0.062

[a]IgM: immunoglobulin M; IgG[b]: immunoglobulin G; IgA[c]: immunoglobulin A; C3[d]: complement C3

Immunofluorescence score data was not normally distributed and statistical comparisons were performed using Kruskal-Wallis analysis, followed by the Dunns' post-hoc test. Significance was set at $p<0.05$. Calculations were performed using JMP® software.
